# Cryptogenic stroke, left atrial function, and atrial fibrillation: a complex relationship

**DOI:** 10.1007/s00392-025-02743-z

**Published:** 2025-09-02

**Authors:** Francesca Augusta Gabrielli, Gianluigi Bencardino, Antonio Di Renzo, Serena Abruzzese, Francesca Colò, Pasquale Alessandro Festa, Lorenzo Severo, Federico Ballacci, Gaetano Antonio Lanza, Antonella Lombardo, Gemma Pelargonio, Aldobrando Broccolini

**Affiliations:** 1 Cardiology Department, Fondazione Policlinico Universitario A. Gemelli IRCCS, Rome, Italy; 2 Cardiology Department, IRCCS Humanitas Research Hospital, Via Alessandro Manzoni, Milan, Italy; 3 Department of Neuroscience, Catholic University School of Medicine, Rome, Italy; 4 Center for Experimental Neurology, Department of Neurology, Bern University Hospital and University of Bern, Bern, Switzerland; 5 Cardiology Department, Università Cattolica del Sacro Cuore, Fondazione Policlinico Universitario A. Gemelli IRCCS, Rome, Italy; 6 Cardiothoracic Department, University Hospital Santa Maria Della Misericordia, Udine, Italy; 7 Neurology Unit, Fondazione Policlinico Universitario A. Gemelli IRCCS, Rome, Italy

**Keywords:** Stroke, Atrial fibrillation, Speckle tracking echocardiography, Implantable loop-recorder

## Abstract

**Background:**

Paroxysmal atrial fibrillation (AF) may underlie some embolic strokes of undetermined source (ESUS), but the widespread use of loop recorders (LRs) to detect it may not be cost-effective. This study evaluated whether assessing left atrial (LA) function by speckle tracking echocardiography (STE) could help to identify ESUS patients most likely to benefit from LR monitoring for AF detection.

**Methods:**

Consecutive ESUS patients diagnosed between 2020 and 2023, who underwent LR implantation and comprehensive echocardiographic evaluation, including STE, were enrolled. Patients were divided into two groups based on AF detection by LR over a median follow-up of 10.0 months (IQR 6.0–21.7).

**Results:**

A total of 64 patients were included: 27 (42.2%) with AF (AF group) and 37 (57.8%) without AF (No-AF group). Compared to the No-AF group, patients in the AF group showed a significantly larger left atrial volume index (LAVi: 44.7 ± 10.8 vs. 34.4 ± 8.3 mL/m^2^; *p* < 0.001), a lower LA longitudinal strain of reservoir (LASr: 19.7 ± 8.9% vs. 27.4 ± 9.5%; *p* = 0.003) and contraction (LASct: 7.4 ± 6.5% vs. 12.4 ± 7.2%; *p* = 0.008), and an increased LA stiffness index (LASi: 0.6 ± 0.3 vs. 0.3 ± 0.2; *p* < 0.001). In multivariable Cox regression analysis, only LAVi and LASct remained independent predictors of AF.

**Conclusions:**

LAVi and LASct appear useful and reliable predictors of AF occurrence during follow-up in ESUS patients and may aid in selecting those who are most likely to benefit from LR implantation.

**Graphical abstract:**

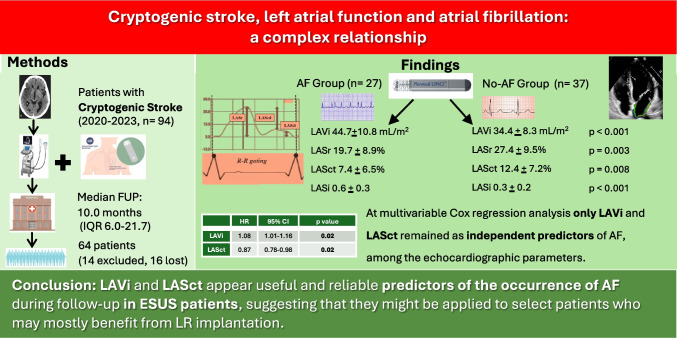

## Introduction

Stroke is a prominent contributor to global mortality, accounting for more than 7 million deaths in 2020 and leading to significant long-term sequelae [1, 2]. However, in about 35% of embolic strokes, no specific cause can be detected, a condition classified as embolic stroke of undetermined source (ESUS) [3, 4]. Previous studies based on long-term heart rhythm monitoring showed that undetected paroxysmal episodes of atrial fibrillation (AF) are present in about 27% of these patients [5]. However, while paroxysmal AF may be hypothesized in patients who have some form of structural heart disease, in several cases, patients with ESUS do not show any apparent cardiac abnormality. Nonetheless, most of them may present abnormalities of atrial myocardial function, suggesting the presence of a subclinical form of atrial cardiomyopathy (AC) [6]. Importantly, strong evidence suggests that besides being a substrate for AF development, AC constitutes an intrinsically prothrombotic substrate that may promote thromboembolic events independently of atrial arrhythmia episodes [7]. Accordingly, recognizing patients who present subclinical AC among those with ESUS might help select those at increased risk for recurrent stroke and in whom heart rhythm monitoring might strongly be indicated to identify the occurrence of paroxysmal AF and subsequent appropriate therapeutic management.

Echocardiography is the reference diagnostic method to identify patients with evidence of AC [8]. Two-dimensional speckle tracking echocardiography (STE), indeed, was shown to be able to assess left atrium (LA) function with high accuracy and reproducibility [9]. To date, several studies have examined LA function and its relationship with AF, showing diminished reservoir, conduit, and contraction functions [10–13] in the occurrence, progression, and recurrence of AF [14, 15].

The aim of our study was to investigate whether impaired LA strain parameters could be associated with a higher prevalence of AF episodes detected by loop recorder during follow-up of patients with ESUS.

## Methods

The prospective database of our comprehensive stroke center was screened for consecutive patients with ESUS who were admitted between 2020 and 2023 and implanted with a loop-recorder for long-term heart rhythm monitoring. All patients underwent a non-contrast computed tomography (CT) scan followed by a cerebrovascular CT angiography to detect the possible occlusion of large-caliber or medium-caliber intracranial arterial vessels. Demographics, vascular risk factors, and baseline clinical features were collected and CHA_2_DS_2_–VASc score was calculated, according to guidelines [16]. Stroke severity at admission was assessed with the National Institute of Health Stroke Scale (NIHSS). Acute reperfusion therapies were performed according to standard protocols [17]. During the hospital stay, patients underwent extensive diagnostic vascular and cardiac studies and received continuous heart rhythm monitoring. Brain magnetic resonance imaging (MRI) was performed in all patients unless contraindicated. Data on location of the ischemic lesion(s) (cortical vs. subcortical) were collected using the available neuroimaging tests. Exclusion criteria were prior history of paroxysmal AF, concurrent conditions requiring anticoagulation (e.g. mechanical prosthetic valves), cardiomyopathies, moderate to severe valve heart disease, more than mild mitral annular calcification, presence of other known cardioembolic sources (e.g. aortic atheroma), and suboptimal echocardiographic windows. Loop recorder implantation was considered for all cases defined as ESUS, after exclusion of large artery atherosclerosis, non-arrhythmic sources of cardio-embolism, and other less common causes of stroke [18].

### Echocardiographic assessment

All patients underwent echocardiographic examination before discharge using commercially available ultrasound machines equipped with a 3.5 MHz phased array probe. The echocardiographic exams were performed by an experienced sonographer or cardiologist.

Standard echocardiographic parameters were measured according to current international recommendations [19, 20]. LA volume (LAV) was indexed for body surface area (LAVi). Among parameters for evaluating diastolic function, E velocity by trans-mitral pulsed-Doppler (PW-Doppler) and e′ velocity (average value between septal and lateral side) of the mitral annulus by pulsed tissue Doppler (PW-TDI) were measured, and E/e′ ratio was calculated.

Data sets were digitally stored and exported to a remote workstation for offline analysis.

After standard examination, two-dimensional strain imaging was performed. All strain parameters were assessed using STE by an external commercially available software program (TomTec Imaging Arena, Unterschleissheim, Germany). Strain analysis was performed using the four-chamber view, according to EACVI guidelines [20].

**Fig. 1:**
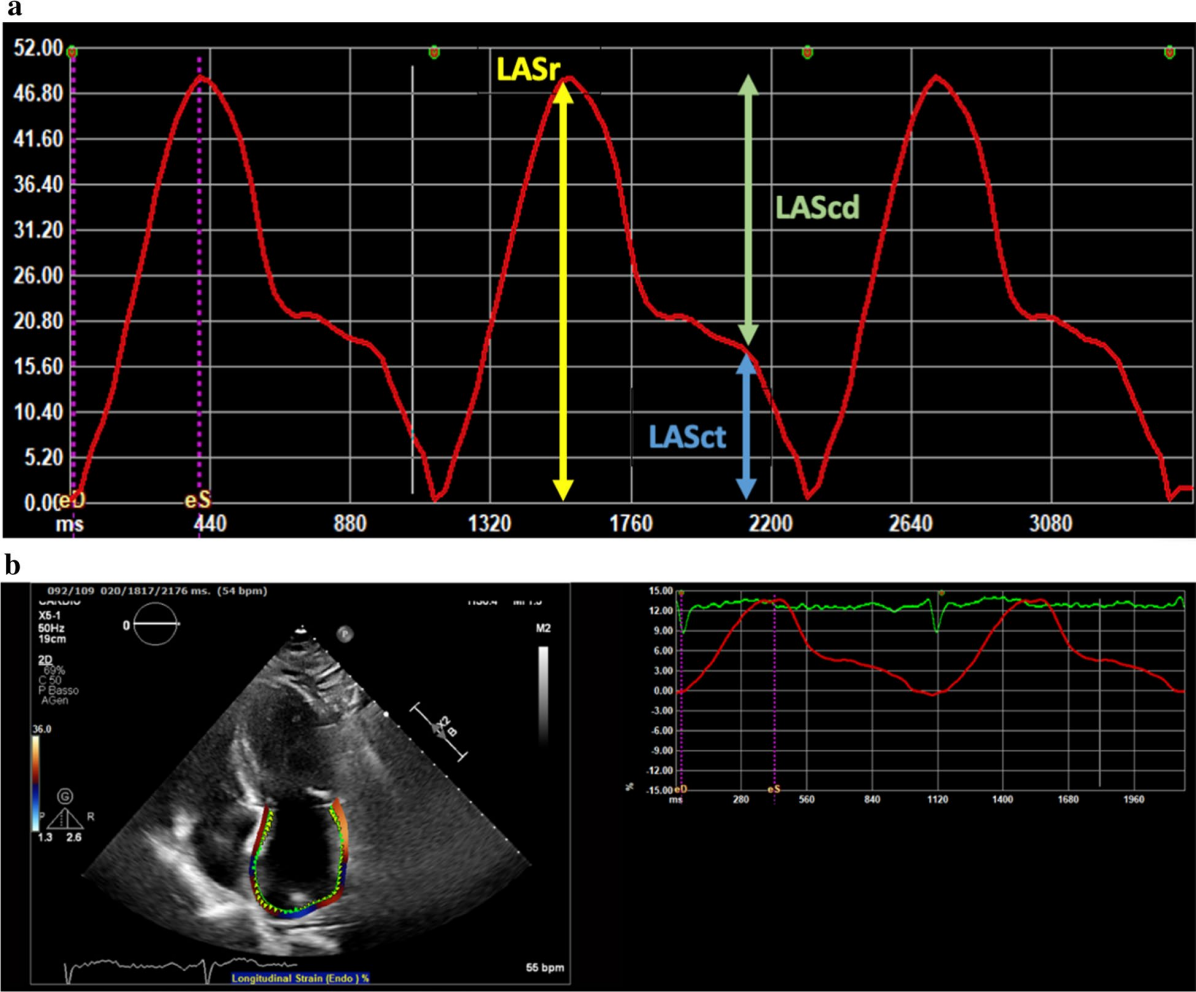
LA strain parameters. **A** Illustration about LA strain phases. **B** Example of left atrial strain measurement. On the left, apical four-chamber view with ROI. On the right, LA strain curves. ED, end-diastolic. ES, end-systolic. For other abbreviations see the text

The region of interest (ROI) was selected using the point-and-click method and manually adjusted to outline the atrial wall, extrapolated across pulmonary veins and atrial appendage, which were excluded to ensure the reliability of automatic calculations. The zero-reference point for image analysis was taken at the onset of the QRS complex (R-R gating), corresponding to the ventricular end-diastole. Thus, the first positive peak was analyzed as LA reservoir strain (LASr, %). The second peak was analyzed as LA contraction strain (peak atrial contraction strain, LASct %). LA conduit strain (LAScd, %) was calculated as the difference of the strain value at the onset of atrial contraction minus mitral valve opening (Fig. 1). LA stiffness index (LASi) was finally assessed by the ratio of E/e′ to LASr.

### Loop recorder implant and follow-up

All patients underwent loop recorder implantation (Reveal LINQ™, Medtronic) before discharge. The implantable loop recorder (ILR) is a miniaturized, subcutaneous device for continuous electrocardiographic monitoring.

Patients were evaluated in the outpatient clinic on a regular basis of 6 months. Additional control visits were performed in case of new symptoms. Data derived from loop recorders were collected using a remote monitoring platform (careLink) and reviewed by expert electrophysiologists. Atrial fibrillation episodes were diagnosed if they lasted more than 30 s, either symptomatic or automatically registered by the device in absence of any referred symptom.

Patients were then divided into two groups based on the detection of AF episodes during follow-up monitoring (AF group and No-AF group).

### Statistical analysis

Data are reported as mean ± standard deviation or median with interquartile range (IQR) for continuous variables and number of subjects and proportions (%) for categorical variables.

The Kolmogorov–Smirnov test was applied to test the normal distribution of continuous variables, which were then compared by the *t* test or the Mann–Whitney test, as indicated. Categorical variables were compared by the Fisher exact test.

Multivariable Cox regression was then applied to identify variables independently associated with AF occurrence, including in multivariable analysis variables with a *p* value < 0.1 at univariate analysis. A *p* value < 0.05 was considered as statistically significant. Statistical analysis was performed using SPSS software ver.22.0 (IBM, Armonk, NY, USA).

Our study has been approved by our ethics committee and was therefore conducted in accordance with the ethical standards set out in the 1964 Declaration of Helsinki and its later amendments. Relevant national laws were also observed.

## Results

During the study period, 94 patients with ESUS underwent LR monitoring. Of these, 14 (14.9%) were excluded due to the presence of exclusion criteria; furthermore, 16 patients (17%) were lost to follow-up. Thus, 64 patients eventually constituted our study population (Tables 1 and 2).

**Table 1:** Clinical characteristics of study population

Clinical characteristics	Study population
Age (years, mean ± SD)	70.8 ± 10.9
Gender (females, %)	32 (50)
Previous stroke (*n*, %)	8 (12.5)
Hypertension (*n*, %)	47 (73.4)
Smoke (*n*, %)	15 (23.4)
Diabetes (*n*, %)	9 (14.1)
Dyslipidemia (*n*, %)	20 (31.3)
CAD (*n*, %)	4 (6.3)
Carotid atherosclerosis (*n*, %)	5 (7.8)
COPD (*n*, %)	6 (9.4)
LVO (*n*, %)	15 (23.4)
Acute cortical lesions (*n*, %)	49 (76.6)
Antiplatelet therapy (*n*, %)	24 (37.5)
Statins (*n*, %)	19 (29.7)
Beta-blockers (*n*, %)	22 (34.3)
CHA_2_DS_2_ VASc Score (mean ± SD)	4.8 ± 1.5
NIHSS admission (mean ± SD)	4.5 ± 4.6
AF at follow-up (*n*, %)	27 (42.2)
Follow-up (median, IQR)	10.0 (6.0–21.7)

*CAD* coronary artery disease, *CHA2DS2-VASc* Congestive heart failure, Hypertension, Age ≥ 75 years, Diabetes mellitus, Stroke, Vascular disease, Age 65–74 years, Sex category (female), *COPD* chronic obstructive pulmonary disease, *LVO* large vessel occlusion, *NIHSS* National Institutes of Health Stroke Scale

**Table 2:** Echocardiographic characteristics of study population

Echo parameters	
EF (%, mean ± SD)	60.7 ± 6.3
LAVi (mL/m^2^)	38.7 ± 10.7
LASr (%)	24.1 ± 9.94
LASct (%)	10.2 ± 7.3
E/e′	8.8 ± 2.5
LASi	0.4 ± 0.2
LAScd (%)	13.9 ± 6.0

*EF* ejection fraction, *LASr* left atrial reservoir strain, *LAScd* left atrial conduit strain, *LASct* left atrial contraction strain, *LASi* left atrial stiffness index, *LAVi* left atrial volume index

During a follow-up of 10 months (IQR 6.0–21.7), episodes of paroxysmal AF were detected with LR monitoring in 27 patients (42.2%; AF Group), whereas no AF episodes were detected in 37 (57.8%; No-AF group). Table 3 shows the main clinical characteristics of the two groups of patients.

**Table 3:** Clinical characteristics of the two groups of patients

	AF group (*n* = 27)	No-AF group (*n* = 37)	*p*
Age (years, mean ± SD)	73.7 ± 7.9	68.7 ± 12.4	0.06
Gender (females *n*, %)	16 (59.3)	16 (43.2)	0.54
previous stroke (*n*, %)	6 (22.2)	2 (5.4)	< 0.001
Hypertension (*n*, %)	21 (77.8)	26 (70.3)	0.45
Smoke (*n*, %)	5 (18.5)	10 (27.0)	0.38
Diabetes (*n*, %)	4 (14.8)	5 (55.6)	0.39
Dyslipidemia (*n*, %)	8 (29.6)	12 (32.4)	0.93
CAD (*n*, %)	3 (11.1)	1 (2.7)	0.06
Carotid atherosclerosis (*n*, %)	3 (11)	2 (5.4)	0.11
COPD (*n*, %)	4 (14.8)	2 (5.4)	0.35
LVO (*n*, %)	10 (37)	5 (13.5)	0.09
Acute cortical lesions (*n*, %)	25 (92.6)	24 (64.9)	0.05
Previous stroke (*n*, %)	6 (22.2)	2 (5.4)	< 0.001
CHA_2_DS_2_ VASc Score	5.3 ± 1.5	4.4 ± 1.5	0.03
NIHSS admission	5.2 ± 4.8	3.9 ± 4.4	0.37
Statins (*n*, %)	10 (37)	9 (24.3)	0.19
Antiplatelet therapy (*n*, %)	12 (44.4)	12 (32.4)	0.07
Beta-blockers (*n*, %)	14 (51.9)	8 (21.6)	0.002
Follow-up (median, IQR)	6.0 (3.0–10.0)	19.0 (9.0–35.0)	< 0.001

*CAD* coronary artery disease, *CHA*_*2*_*DS*_*2-*_*VASc* Congestive heart failure, Hypertension, Age ≥ 75 years, Diabetes mellitus, Stroke, Vascular disease, Age 65–74 years, Sex category (female), *COPD* chronic obstructive pulmonary disease, *LVO* large vessel occlusion, *NIHSS* National Institutes of Health Stroke Scale

Compared to those of the No-AF group, patients of AF group had a significantly higher rate of previous stroke (22.2% vs. 5.4%; *p* < 0.001), a higher CHA2DS2-VASc score (5.3 ± 1.5 vs. 4.4 ± 1.5, *p* = 0.03) and were more frequently treated with beta-blocker therapy (51.9% vs 21.6%, *p* = 0.002). AF group patients were also older and showed an increased prevalence of coronary artery disease (CAD) and a higher rate of acute large vessel occlusion (LVO) as the cause of the index stroke; these differences, however, did not achieve statistical significance.

**Fig. 2:**
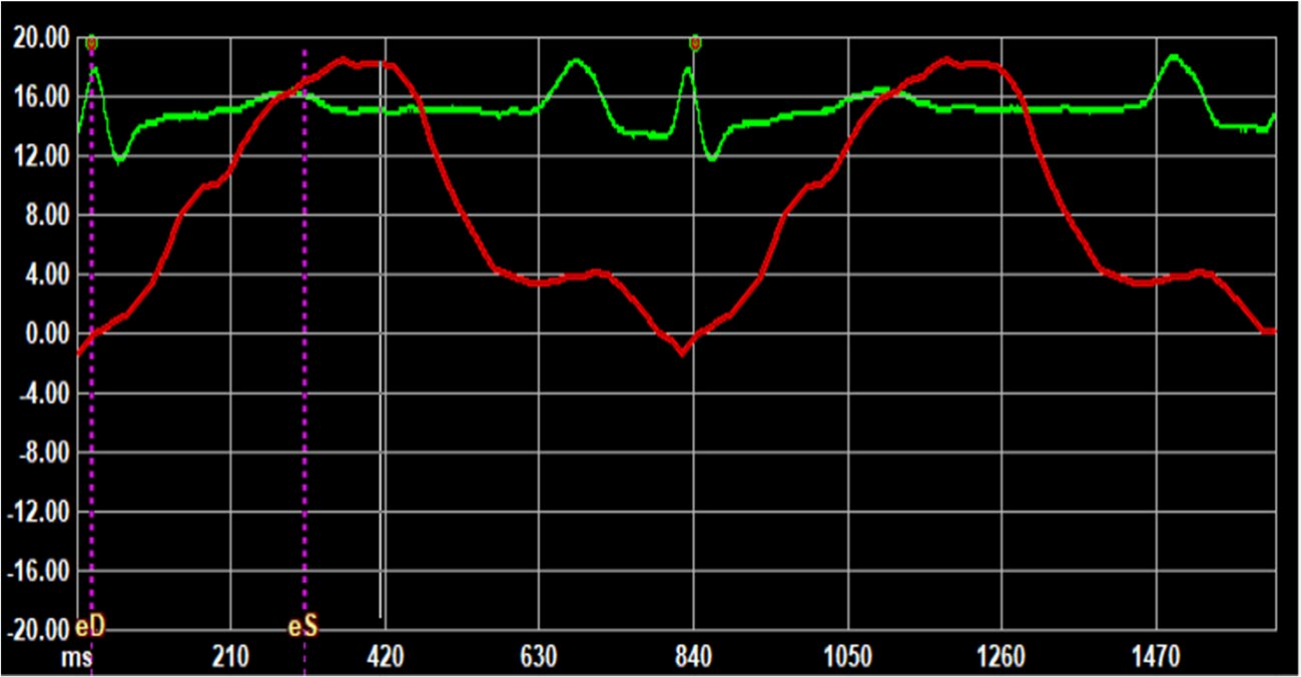
Example of reduced values on left atrial strain, in particular reduction of LASct, in AF-group patient

Table 4 shows the echocardiographic data of the two groups of patients. Patients in AF group, compared to those of No-AF group, showed an increased E/e′ ratio (9.9 ± 2.8 vs. 8.0 ± 1.9, *p* = 0.007) and a larger LA volume index (LAVi, 44.7 ± 10.8 vs. 34.4 ± 8.3 mL/m^2^; *p* < 0.001). Moreover, all LA strain parameters and LASi were significantly reduced in patients of AF group compared to those of No-AF group (Fig. 2).

**Table 4:** Main echocardiographic parameters in the two groups of patients

	AF group (*n* = 27)	No-AF group (*n* = 37)	*p*
EF (%, mean ± SD)	59.1 ± 7.3	61.8 ± 5.3	0.05
LAVi (mL/m^2^)	44.7 ± 10.8	34.4 ±.8.3	< 0.001
LASr (%)	19.7 ± 8.9	27.4 ± 9.5	0.003
LASct (%)	7.4 ± 6.5	12.4 ± 7.2	0.008
E/e′	9.9 ± 2.8	8.0 ± 1.9	0.007
LASi	0.6 ± 0.3	0.3 ± 0.2	< 0.001
LAScd (%)	12.3 ± 6.0	15.0 ± 5.9	0.15

*EF* ejection fraction, *LAVi* left atrial volume index, *LASi* left atrial stiffness index, *LASr* left atrial reservoir strain, *LASct* left atrial contraction strain, *LAScd* left atrial conduit strain

The results of multivariable survival Cox regression analysis are reported in Table 5. Of clinical variables, only a prior history of stroke (*p* < 0.001) and evidence of a recent stroke due to large vessel occlusion (*p* = 0.002) remained strongly associated with an increased odds of AF occurrence during LR monitoring. A history of CAD was also associated with a reduced odds of AF, although with less strength. On the other hand, patients with beta-blocker therapy showed an increased odds of AF. Finally, among echocardiographic parameters, only LAVi (*p* = 0.02) and LASct (*p* = 0.02) remained independent predictors of the occurrence of AF during follow-up.

**Table 5:** Multivariable analysis of clinical and echocardiographic parameters independently associated with atrial fibrillation (AF) occurrence

	HR	95% CI	*p* value
LVO	6.86	2.0–23.3	**0.002**
Age	0.97	0.8–1.05	0.43
Previous stroke	18.41	4.18–81.14	** < 0.001**
CAD	0.03	0.002–0.41	**0.009**
Antiplatelet therapy	2.33	0.70–7.80	0.17
Beta-blocker therapy	3.6	1.16–11.17	**0.03**
Acute cortical lesions	2.7	0.50–14.55	0.25
CHA_2_DS_2_ VASc Score	1.4	0.82–2.57	0.2
EF	0.94	0.88–1.00	0.09
LAVi	1.08	1.01–1.16	**0.02**
E/e′	1.13	0.81–1.58	0.44
LASr	0.99	0.87–1.14	0.93
LASct	0.87	0.78–0.98	**0.02**
LASi	0.11	0.002–6.72	0.29

*CAD* coronary artery disease, *CHA*_*2*_*DS*_*2-*_*VASc*: Congestive heart failure, Hypertension, Age ≥ 75 years, Diabetes mellitus, Stroke, Vascular disease, Age 65–74 years, Sex category (female), *EF* ejection fraction, *LAVi* left atrial volume index, *LVO* large vessel occlusion, *LASi* left atrial stiffness index, *LASr* left atrial reservoir strain, *LASct* left atrial contraction strain, *LAScd* left atrial conduit strain

## Discussion

In this study, we found that patients with ESUS, who were subsequently found to have AF episodes at LR during follow-up, had evidence of unfavorable LA remodeling and impaired LA function, as assessed by STE performed during the index cerebral ischemic attack. Specially, we found that LASct, an expression of atrial kick, was significantly related to the occurrence of AF in this population, suggesting that in patients with ESUS, the detection of an abnormal LASct may predict AF episodes at follow-up.

Stroke is a major cardiovascular disease with high mortality and morbidity, and AF is responsible for up to one-third of all ischemic strokes [21]. In particular, paroxysmal AF is a major cause of ESUS, but it remains undetected in a high proportion of patients when it is no longer present at the time of hospital admission [22].

Although standard echocardiography may suggest AF as a possible cause of ESUS by showing LA enlargement or significant heart disease, the predictive accuracy of these findings is limited [23]. Technical progresses in echocardiography now allow reliable assessment of atrial myocardial function, the impairment of which might favor the occurrence of AF [24].

Importantly, there is increasing evidence that atrial dysfunction might by itself be associated with an increased risk of thromboembolism, independently of AF [25, 26]. Some studies, indeed, failed to find a temporal association between AF episodes and stroke events during LR monitoring, suggesting that an atrial “cardiomyopathy” might by itself be responsible for the thromboembolic risk [27, 28]. Thus, the exact mechanisms by which LA dysfunction contributes to the occurrence of thromboembolic events remain not fully understood.

The data of the present study support an AF-mediated relation with ESUS of structural and functional alterations of LA. Patients who were found to develop AF episodes during follow-up, indeed, had a significant greater LA enlargement compared to those who did not show any occurrence of AF during follow-up, a finding in agreement with the results of previous studies [29].

The mechanism by which LA enlargement favors AF mainly resides on the fact that it is frequently associated with increased atrial fibrosis [30, 31], which facilitates the occurrence of a disorganization of electrical activity, typical of AF. The association of LA enlargement with fibrosis is supported, in our study, by the impairment of all LA atrial strain parameters, especially those related to LA booster function and stiffness.

These results are also in agreement with several previous studies that reported an association between an impairment of both systolic and diastolic LA function with increased rates of AF [32–34].

The fact that functional atrial abnormalities may act as thromboembolic risk factors mainly through the predisposition to AF is also supported in our study by the more significant impairment of the indices of LA function in patients who developed AF compared to those who did not develop AF during follow-up.

Notably, at multivariable analysis the peak atrial contraction phase (LASct), in agreement with previous studies [24, 29, 32, 33], maintained a strong independent correlation with AF detection at follow-up, thus suggesting that it can be a major marker or component of the arrhythmogenic risk related to LA abnormalities. LASct, indeed, most likely reflects the presence of LA fibrosis and remodeling [35], which eventually favor AF occurrence.

The originality of our study, compared to others, is that we found a stronger relationship between AF detection and LA contraction than reservoir strain, suggesting that it might be a more specific parameter related to mechanical and consequently electric atrial function.

We should be aware, however, that our data do not allow to conclude that the stronger abnormal atrial function in patients with evidence of AF exerts its arrhythmogenic effect through the induction of the arrhythmia, as it might independently cause both an increased risk of AF and thromboembolic events. Accordingly, we cannot completely exclude that an impairment of minor degree of atrial function might also constitute a risk factor for thromboembolic stroke, independently of the occurrence of AF, as we did not assess atrial function in a comparable group of healthy control subjects.

Our data also show an independent association of LVO and a history of prior stroke with AF occurrence during LR monitoring, which is also in agreement with previous findings, further supporting the established evidence [36, 37].

Previous studies investigated the relationship between AF and CAD [38, 39]. Unexpectedly, CAD was not associated to the risk of AF in our study. This result might be related to the small number of the study population. However, another possible explanation may be that patients with CAD were strictly followed and assumed a more broad-spectrum therapy, active on the same comorbidities associated to AF development [40]. On the other hand, beta-blocker therapy was found associated to an increased odd of AF. In this case, it is possible that patients took beta-blockers for a history of palpitations, probably symptom of an unacknowledged paroxysmal AF instead.

The most relevant clinical implication of our data is that among patients with ESUS, specific attention in continuous monitoring for the detection of AF should be paid in those who show clear findings of LA functional impairment, in particular a depressed LASct index. In this way, a proper selection of patients for ILR could be pursued, in order to detect as soon as possible patients with PAF, so driving anticoagulation decisions, to reduce avoidable complications and to optimize public health resources.

### Limitations of the study

Some limitations of our study should be acknowledged. First, the retrospective nature of the study introduces a potential selection bias, as loop recorder implantation was performed on cryptogenic stroke patients suspected of having cardio-embolism. Selection for ILR was based on clinical characteristics, and this may have led to the enrollment of patients with more compromised cardiovascular profiles. Second, although clinical and procedural records were carefully reviewed, the results could be influenced by data quality constrains inherent to non-randomized studies. Third, it included only a small number of patients, and therefore, our data need validation in larger prospective cohorts. As noticed above, the lack of a control group of healthy subjects limits the interpretation of our data, in particular as far as whether an abnormal LA function is also present in patients without detection of AF at follow-up. Finally, we analyzed LA strain using only four-chamber view, which might have limited the precision of the assessment; however, we assessed atrial function according to international guideline recommendations [20].

## Conclusions

In patients with ESUS, the detection of abnormalities in LA function at STE, particularly an impaired LASct, compatible with the presence of a subclinical atrial cardiomyopathy, is associated with an increased rate of AF occurrence at LR monitoring, during follow-up, suggesting that the index acute ischemic event was caused by cardiac thromboembolism complicating this arrhythmia. While we cannot exclude an independent role of impaired LA function for stroke occurrence, our data indicate that patients with ESUS who show impaired LASct should undergo continuous monitoring through LR to assess whether AF occurs during follow-up, which can be crucial for an appropriate management of patients.
